# Dysregulated Intestinal Host–Microbe Interactions in Systemic Lupus Erythematosus: Insights from Patients and Mouse Models

**DOI:** 10.3390/microorganisms13030556

**Published:** 2025-03-01

**Authors:** Miki Kume, Jin Din, Daniel F. Zegarra-Ruiz

**Affiliations:** Carter Immunology Center, University of Virginia, Charlottesville, VA 22908, USA; kume@virginia.edu (M.K.); uwc8jz@virginia.edu (J.D.)

**Keywords:** systemic lupus erythematosus, autoimmunity, intestinal microbiota, intestinal barrier impairment, intestinal dysbiosis, pathobionts

## Abstract

Systemic lupus erythematosus (SLE) is an autoimmune disease characterized by chronic inflammation that affects multiple organs, with its prevalence varying by ethnicity. Intestinal dysbiosis has been observed in both SLE patients and murine models. Additionally, intestinal barrier impairment is thought to contribute to the ability of pathobionts to evade and breach immune defenses, resulting in antigen cross-reactivity, microbial translocation, subsequent immune activation, and, ultimately, multiple organ failure. Since the detailed mechanisms underlying these processes are difficult to examine using human samples, murine models are crucial. Various SLE murine models, including genetically modified spontaneous and inducible murine models, offer insights into pathobionts and how they dysregulate systemic immune systems. Furthermore, since microbial metabolites modulate systemic immune responses, bacteria and their metabolites can be targeted for treatment. Based on human and mouse research insights, this review examines how lupus pathobionts trigger intestinal and systemic immune dysregulation. Therapeutic approaches, such as fecal microbiota transplantation and dietary adjustments, show potential as cost-effective and safe methods for preventing and treating SLE. Understanding the complex interactions between the microbiota, host factors, and immune dysregulation is essential for developing novel, personalized therapies to tackle this multifaceted disease.

## 1. Introduction

Dysbiosis, a disruption in intestinal microbial balance, has been implicated in the pathogenesis of various diseases, including autoimmune conditions like multiple sclerosis, type 1 diabetes, and systemic lupus erythematosus (SLE) [1]. SLE is a multifaceted autoimmune disease characterized by chronic inflammation and the production of autoantibodies, affecting multiple organ systems [2,3]. While the precise etiology of SLE remains unclear, dysbiosis is emerging as a potential environmental factor [4,5,6,7,8]. Metagenomic analyses and 16S rRNA sequencing have revealed dysbiosis and its association with immune dysfunction in SLE patients. Additionally, research using human blood and peripheral organ samples provides insights into how pathobionts affect human immune systems. To further understand these mechanisms, research on murine models is crucial. Various lupus models have been utilized to analyze host–microbe interactions in lupus. These studies involve examining dysbiosis and antibiotic treatments and assessing specific conditions, such as specific-pathogen-free (SPF), germ-free (GF), and monocolonized states with specific pathobionts. Over the past decade, numerous pathways in which pathobionts contribute to lupus pathogenesis have been identified, including microbial translocation and antigen mimicry, demonstrating how specific microbes interfere with intestinal and systemic immune systems.

Understanding how commensals acquire pathobiont characteristics and disrupt immune processes beyond the intestine offers essential insights into the etiology and treatment of lupus. This review examines how lupus-associated pathobionts influence the intestinal milieu and alter systemic immune processes, as studies in human and murine lupus models demonstrate. Furthermore, we describe four intestinal bacteria whose pathogenetic roles in lupus have been well explored: *Enterococcus gallinarum*, *Ruminococcus gnavus*, *Bacteroides thetaiotaomicron*, and *Lactobacillus reuteri*. Additionally, we discuss potential future therapeutic approaches to modulate these interactions.

## 2. Systemic Lupus Erythematosus Immunobiology and Etiopathogenesis

SLE is a chronic, heterogeneous, multifactorial, and multiorgan-mediated autoimmune disease with risks including genetic variations, environmental stressors, and abnormalities in the immune system [2,9]. Renal disease is a significant contributor to the mortality of SLE. It often develops early, with more than half of patients developing lupus nephritis within five years of SLE onset [10,11,12]. The manifestations of lupus nephritis range from asymptomatic proteinuria and hematuria to end-stage renal disease, which affects up to 20% of patients [12]. The histopathology of lupus nephritis is classified into six classes based on the absence of detectable changes by light microscopy but with immunodepositions (class I), mesangial hypercellularity (class II), endocapillary hypercellularity (class III: focal, class IV: diffuse), or membranous changes (class V) [12,13]. This classification is crucial for diagnosis and treatment decisions. Although treatment effectiveness and tolerance vary among ethnicities and guidelines tailor treatment recommendations to the populations they are intended for, hydroxychloroquine is broadly considered an anchor drug. For class III/IV ± V lupus nephritis, glucocorticoids combined with either mycophenolate mofetil or cyclophosphamide are used as first-line therapy [11]. Arthritis and cutaneous lupus erythematosus (CLE) often appear as initial symptoms. Arthritis and arthralgias are initial symptoms in 57.6% and 20.3% of patients, respectively, affecting up to 48.1–88.1% during the disease progression [14,15]. CLE can manifest as an isolated skin condition or as an SLE symptom. Approximately 70–80% of SLE patients exhibit skin symptoms, and in 20–25% of cases, skin manifestations are the initial presentation of SLE [16]. Additionally, neuropsychiatric SLE and lupus myocarditis can be life-threatening [17,18,19,20].

SLE pathogenesis involves innate and adaptive immune responses, with various cell types and cytokines playing essential roles. Type Ⅰ interferon (IFN), represented by IFN-α, is thought to play a key role in SLE pathogenesis [18,21]. In plasmacytoid dendritic cells (pDCs), aberrant recognition of self-antigens occurs, such as single-stranded RNA by endosomal Toll-like receptor (TLR)7 and unmethylated CpG DNA by TLR9, which induces IFN-α expression. While the number of circulating pDCs decreases in SLE [22,23], pDCs infiltrate peripheral organs and cause local inflammation through IFN production, as observed in the skin [24] and renal tissue [22]. Increased levels of type Ⅰ IFNs promote multiple processes that support lupus pathogenesis [23,25,26,27].

T-cell activation and differentiation are also dysregulated in SLE. The phenotypes of helper T (Th) cells, including Th1, Th2, Th17, regulatory T cells (Tregs), follicular helper T (T_FH_) cells, and TCRα^+^CD3^+^CD4^−^CD8^−^ double-negative (DN) T cells are altered in SLE [28]. Among the central cytokines produced by Th1, Th2, and Th17 cells—IFN-γ, IL-4, and IL-17A, respectively—the role of IL-17A, which promotes the recruitment of inflammatory cells to peripheral organs, has been studied in detail in SLE. Serum levels of IL-17A are upregulated, and the numbers of Th17 cells and DN T cells, which are efficient producers of IL-17A in SLE, are increased [29,30,31]. Serum levels of IFN-γ, mainly produced by Th1 cells, cytotoxic CD8^+^ cells, and NK cells, correlate with disease activity [32]. IFN-γ stimulates antigen-presenting cells to produce B-lymphocyte stimulating factor (BLyS) and promotes the formation of germinal centers [32]. Furthermore, IFN-γ enhances the B-cell differentiation into antibody-secreting cells [33]. On the contrary, the changes in the number and function of Tregs are controversial [34]. Tregs can be impaired in peripheral organs even if changes in their numbers and functions in circulation are not observed [35]. This indicates that the microenvironment is crucial for T-cell functions. Antigen-presenting cells are crucial for naïve T-cell activation and antibody production from B cells [36]. T_FH_ cells support various functions within the germinal center, including B-cell survival, proliferation, plasma cell differentiation, hypermutation, immunoglobulin class switching, adhesion, and attraction [37]. Circulating T_FH_ (cT_FH_) cells are the blood counterparts of T_FH_ cells and are essential for plasmablast differentiation [38]. T_FH_ and cT_FH_ cells express CXCR5, enabling them to enter B-cell follicles. T peripheral helper (T_PH_) cells can also activate B cells despite lacking CXCR5 expression [39]. In circulation, type Ⅰ IFN promotes the accumulation of CXCL13^+^ T_FH_/T_PH_ cells by counteracting the roles of aryl hydrocarbon receptor (AHR), AP-1 family member JUN, and IL-2. These T_FH_/T_PH_ cells can support B-cell activity in SLE within peripheral tissues and circulation [40].

B cells produce autoreactive antibodies in autoimmune diseases. Anti-double-strand (ds)DNA and anti-Sm antibodies are specific to SLE. They are included in the 2019 EULAR/ACR classification criteria for SLE [41]. Anti-Ro antibodies can be found in SLE and Sjögren’s syndrome, systemic sclerosis, and myositis [42]. However, they are noteworthy because they appear earlier than other SLE-related autoantibodies, such as anti-dsDNA and anti-Sm antibodies, and are present an average of 3.4 years before an SLE diagnosis [43]. Epitope spreading by B-cell/T-cell interactions contributes to the amplification of SLE-related autoantibodies [44]. These antibodies form immune complexes with their respective antigens, depositing in peripheral organs and causing inflammation.

SLE exhibits significant variation in its prevalence and incidence based on sex and ethnicity. It affects women six to nine times more than men in the US and is particularly common among women of childbearing age [45,46]. Sex hormones and sex chromosomes that encode *TLR7* and *TLR8* are thought to contribute to the high prevalence of SLE in women [47,48]. Additionally, SLE in the US is more prevalent among African Americans, followed by Native Americans, with intermediate levels in Asians and Hispanics, and the lowest prevalence is observed in Whites. The incidence follows a similar pattern, with the highest rates in African Americans and Native Americans [45]. Ethnicity also influences autoantibody profiles. For example, patients of African American ancestry are more likely to be positive for both anti-dsDNA and anti-RNP antibodies than those of European ancestry [49]. Additionally, since there is significant diversity within the ‘Hispanic’ category, the phenotypes of SLE can vary among Hispanic populations. The Latin American Group for the Study of Lupus (GLADEL) original cohort conducted a detailed study on this diversity. They classified individuals from Latin American countries, often broadly labeled as Hispanic, into three groups: Mestizo, Caucasian, and African Latin American (ALA). It was found that Mestizos and ALAs showed a statistically higher likelihood of developing lymphopenia, and Mestizos were more prone to renal damage compared to Whites [45,46]. These variations in prevalence indicate that the pathogenesis and phenotypes of SLE highly depend on genetic background. Furthermore, the global prevalence of SLE has been increasing over time [50,51], which is supposed to be influenced by environmental exposures, dietary habits, healthcare accessibility, and socioeconomic inequities.

Recently, there has been growing interest in the relationship between alterations in the intestinal microbiome and autoimmune diseases, such as rheumatoid arthritis, multiple sclerosis, type 1 diabetes mellitus, and SLE [1]. Ethnic and sex biases in the intestine microbiome have been reported [52,53,54]. Ethnicity reflects not only genetic background but also comprehensive factors such as dietary habits, the prevalence of specific diseases, hygiene status, and other environmental influences. The association between microbiota and genetic background indicated by ancestries is thought to be limited [55]. These findings indicate an interaction between SLE pathogenesis and the microbiome. Since diet influences the intestinal microbiome [54,55], dietary interventions are considered an attractive approach to modulating disease development.

The microbiota and host immune cells are in constant interaction within the intestine, with the host’s health depending on a fragile equilibrium between them. When this balance is disturbed, microbes may initiate or exacerbate autoimmune responses in the host. The following section will examine host–microbe interactions seen in SLE patients.

## 3. Intestinal Dysbiosis in Systemic Lupus Erythematosus

The human large intestine harbors 10^11^ to 10^12^ bacteria per gram of colonic content in a healthy state, much larger than the stomach, duodenum, and jejunum/ileum [56]. Consequently, large intestine bacteria contribute to about 60% of the fecal mass [56]. The healthy intestinal microbiota plays a symbiotic role with the host and contributes to food fermentation, metabolism, and immune system development [57]. The microbiota protects the host from invading pathogens and contributes to immune system signaling through bacterial metabolites, bacterial components, and the bacteria themselves, as well as by promoting the formation of robust mucosal barriers [58]. Recently, impaired intestinal barrier functions and dysbiosis have been reported in SLE patients [4,6], and some mechanisms by which pathobionts are associated with SLE pathogenesis have been elucidated. Studies on intestinal dysbiosis in SLE patients are primarily conducted on fecal samples using 16S rRNA sequencing due to its cost-effectiveness and scalability for larger projects [59]; 16S rRNA sequencing typically categorizes microbes down to the genus level. In some cases, species-level resolution can be achieved, especially with full-length 16S gene sequencing and advanced analysis methods [60,61]. This method mainly allows for phylogenetic analysis without providing functional insights. The 16S rRNA sequencing of IgA-coated microbiota (IgA-seq) helps identify immunomodulatory microbes [62]. This is because IgA is the most abundant antibody isotype on mucosal surfaces, and high levels of IgA coating indicate bacteria that can selectively stimulate intestinal immunity [62]. Shotgun metagenomic analysis sequences entire microbial genomes and is used to investigate the functions of these microbiomes [59]. Additionally, metagenome-wide association studies (MWAS) can reveal microbiome–disease associations beyond the scope of standard metagenomic analysis [60].

The predominant bacterial phyla in the human intestine are *Firmicutes*, *Bacteroidetes*, *Actinobacteria*, and *Proteobacteria*, with *Firmicutes* and *Bacteroidetes* accounting for more than 90% of the intestinal microbiota [63,64]. In SLE patients, microbiota diversity, as indicated by α-diversity, is significantly reduced compared to healthy controls [6,65,66,67,68,69]. Conversely, β-diversity analysis shows that microbiome composition significantly differs between SLE and healthy subjects [6,68,70]. The *Firmicutes* phylum includes genera such as *Lactobacillus*, *Bacillus*, *Clostridium*, *Enterococcus*, *Staphylococcus*, and *Ruminococcus*. The *Bacteroidetes* phylum primarily comprises genera like *Bacteroides* and *Prevotella* [63]. Various studies have revealed that the *Firmicutes*/*Bacteroidetes* (F/B) ratio is decreased in SLE patients [7,67,69,71], while some studies showed no significant changes in F/B levels between SLE patients and healthy controls [65,68,72]. However, this change is not specific to SLE, as a reduced F/B ratio is also observed in type 2 diabetes mellitus, inflammatory bowel disease, and breast cancer [73,74,75]. Conversely, the F/B ratio is increased in obesity [73]. Therefore, investigating the microbiome’s functions and host–microbe interactions at the species level can help us better understand their roles in disease pathogenesis.

The 16S rRNA sequencing of fecal samples revealed differences in the phyla, families, genera, and species of bacteria between SLE patients and healthy controls (Table 1) and between active and inactive SLE patients [68,69]. Shotgun metagenomic sequencing revealed that *Clostridium* species ATCC BAA-442, *Atopobium rimae*, *Shuttleworthia satelles*, *Actinomyces massiliensis*, *Bacteroides fragilis*, *Clostridium leptum*, and unclassified *Escherichia* are enriched in SLE patients and reduced after treatment [70]. This study and other studies have reported a significant upregulation of *Ruminococcus gnavus* in SLE patients with active disease or lupus nephritis [6,69,70]. Similarly, IgA-seq showed a trend of upregulated *R. gnavus* in SLE patients [6]. MWAS revealed an increase in *Streptococcus intermedius* and *Streptococcus anginosus* in SLE patients, along with a positive correlation between plasma levels of acylcarnitine (18:1), which is known to act as an inflammatory signal, and *Streptococcus intermedius* [60].

In addition to those insights gained from the analysis of fecal samples, examining serological, blood, and tissue samples provides us with more precise knowledge about microbiome-related SLE pathogenesis. The expansion of *R. gnavus* in the intestine and a compromised intestinal barrier leads to cross-reactivity with anti-dsDNA immune responses, thereby influencing the immune complex-driven pathogenesis of lupus nephritis [6,80]. As evidenced by findings indicating intestinal barrier impairment and microbial translocation in liver samples, *Enterococcus gallinarum* is thought to contribute to the pathogenesis of SLE [4,8]. T cells from anti-Ro antibody-positive patients exhibit cross-reactivity with commensal Ro60 ortholog, including *Bacteroides thetaiotaomicron* in the intestine and *Propionibacterium propionicum* on skin and human Ro60 protein [7]. Since anti-Ro antibodies are thought to contribute to epitope spreading [81], microbes with Ro60 orthologs are worth investigating, particularly for their roles in the initial stages of lupus development.

In addition to intestinal dysbiosis, alterations in the skin, nasal, oral, and vaginal microbiome have also been reported. For instance, anti-Ro60 antibodies are thought to be associated with UV sensitivity. The presence of *P. propionicum*, which carries Ro60 orthologs, in the epidermis and deeper layers than the epidermis in CLE patients may contribute to the symptoms of CLE [7]. In CLE patients, elevated IFN-α levels are observed at baseline and after UV exposure, leading to skin barrier disruption and increased colonization by *Staphylococcus aureus* [79]. Cutaneous lupus disease area and severity index tend to be higher in patients colonized with *S. aureus* in the skin [79]. Nasal colonization of *S. aureus* in SLE patients is also associated with renal failure and anti-dsDNA, anti-Sm, anti-SSB, anti-SSA, and anti-RNP antibodies [82]. Additionally, in the oral microbiota, the bacterial families *Lactobacillaceae*, *Veillonellaceae*, and *Moraxellaceae*, as well as genera including *Veillonella* and *Fusobacterium*, were found to be increased in SLE patients [72,78]. In the vaginal microbiota, thirteen bacterial genera exhibiting differences in abundance between SLE patients and healthy controls were identified [76].

Studies using human samples have reported dysbiosis in SLE, with some also identifying associations between dysbiosis and immune responses through metagenomic analysis. Others have shown how an altered microbial composition can affect the host immune system using blood samples and peripheral organ specimens. Although fecal samples, as well as swabs from the skin, oral cavity, and vagina, can reveal an overall picture of the microbiome components, they cannot conclusively determine which microbes truly impact the host immune system and the mechanisms underlying these changes. To address this knowledge gap, animal models are required to study the microbes responsible for the modulation of specific lupus symptoms and understand how they regulate immune responses.

## 4. Intestinal Dysbiosis in Mouse Models of Lupus

In this section, we describe representative mouse lupus models that have been thoroughly studied to understand the role of host–microbe interactions in lupus pathogenesis. For each mouse model, we focus on disease outcomes in GF conditions or in response to antibiotic treatment, detail the changes in microbial communities as the disease progresses, and discuss the immune pathways altered by pathobionts.

### 4.1. NZB/W F1 and NZM2410 Mice

Description of the Model: NZB/W F1 mice are among the oldest SLE models, created by crossing New Zealand Black (NZB) and New Zealand White (NZW) mice. These mice develop lymphadenopathy, splenomegaly, increased antinuclear antibodies, and glomerulonephritis, with a pronounced female bias in disease manifestation [83]. Female mice begin to develop glomerulonephritis at approximately seven months of age [84]. NZM2410 mice, derived from the NZB and NZW strains, exhibit a higher incidence and a more rapid progression of glomerulonephritis, with onset beginning at approximately five months of age [83]. By 12 months of age, up to 80% of both sexes of NZM2410 mice are affected by fatal glomerulonephritis [84,85]. Unlike NZB/W F1 mice, NZM2410 shows a weaker gender bias [84].

Dysbiosis: 16S rRNA sequencing targeting fecal samples from pre-disease onset and post-disease onset in NZB/W F1 mice revealed dynamic changes in the microbiota. Only “*Lactobacillaceae*, *Lactobacillus*, other” significantly increased from pre- to post-disease onset and were decreased by Dexamethasone treatment [65].

Fecal Microbiota Transplantation (FMT) or Colonization Studies: Colonization with segmented filamentous bacteria (SFB) in SPF NZM2410 mice causes changes in intestinal microbiota, including an expansion of *Prevotellaceae*, *Lactobacillaceae*, and *Clostridiaceae*, and a reduction of *Ruminococcaceae* and *Lachnospiraceae* [86]. SFB-colonized NZM2410 mice exhibit exacerbated kidney disease characterized by increased infiltration of F4/80^+^CD206^+^ macrophages, upregulation of proinflammatory cytokines and macrophage chemoattractants in the serum, expansion of Th17 cells and group 3 innate lymphoid cells (ILC3s) in the small intestine, and impaired intestinal barriers compared to NZM2410 mice without SFB colonization [86]. Moreover, 16S rRNA sequencing revealed alterations in the microbiome between SFB-positive and SFB-negative groups, with significant enrichment of *Ruminococcus torques* spp., indicating that SFB colonization can substantially affect the intestinal microbiota [86]. Since C57BL/6J mice do not develop kidney pathology regardless of SFB colonization, a specific genetic background is required to induce intestinal microbiota-associated pathogenesis [86].

### 4.2. MRL/Mp-lpr/lpr (MRL/lpr) Mice

Description of the Model: MRL/lpr mice are a classical model for SLE characterized by lymphadenopathy. In this mouse model, anti-ssDNA, anti-dsDNA, and anti-Sm antibodies are observed, and large amounts of immune complexes cause glomerulonephritis and vasculitis [87,88]. The cognate partner, MRL/Mp mice, develops glomerulonephritis much later than MRL/lpr mice [87]. These phenomena are derived from a mutation in the *fas* gene, which alters the transcription of the Fas receptor and prevents the apoptosis of autoreactive T cells and B-cell clones [89,90]. The MRL genetic background is also essential for the symptoms observed in MRL/lpr mice, as *lpr* mutations in mice with a C3H/HeJ or C57BL/6J background do not lead to renal disease [91]. Although the disease is more accelerated in female MRL/lpr mice, both females and males are affected, with females and males dying at approximately 17 weeks and 22 weeks of age, respectively [87,88,92].

GF or Antibiotics Studies: While GF MRL/lpr mice exhibit a lupus phenotype comparable to conventionally raised mice [93], antibiotic treatment of SPF MRL/lpr mice after disease onset reduces disease severity compared to control MRL/lpr mice [94]. Conversely, early and short-term antibiotic treatment not only caused dysbiosis and subsequent exacerbation of the disease phenotype but also impaired the effectiveness of prednisone therapy [95].

Dysbiosis: MRL/lpr mice show a decrease in *Lactobacillaceae* and an increase in *Lachnospiraceae* compared to MRL/Mp mice [96]. Retinoic acid, a metabolite of vitamin A, can restore *Lactobacillaceae*, prevent the expansion of *Lachnospiraceae*, and improve lupus symptoms [96]. Inoculation with a mix of five *Lactobacillus* species (*Lactobacillus oris*, *Lactobacillus rhamnosus*, *Lactobacillus reuteri*, *Lactobacillus johnsonii*, and *Lactobacillus gasseri*) in SPF MRL/lpr mice improves the lupus phenotype compared to the control MRL/lpr mice [97,98]. It was demonstrated that these species synergistically exert their protective effects, but the microbes cannot achieve the same effect individually [97,98].

FMT or Colonization Studies: Inoculation with a mix of five *Lactobacillus* species improves lupus symptoms, including lupus nephritis, serum autoantibody levels, and mortality in MRL/lpr mice [98]. *Lactobacillus* species also improve splenomegaly and lymphadenopathy through secreted factors and a CX_3_CR1-dependent pathway [98].

### 4.3. TLR7-Dependent Lupus Models

Description of the Model: TLR7-dependent lupus models are divided into spontaneous and inducible models. One of the spontaneous TLR7-dependent lupus models is the transgenic mouse overexpressing TLR7 in a C57BL/6J background (TLR7.1 Tg) [99]. The motivation for focusing on TLR7 in mouse models originates from the observation that almost all male BXSB mice develop lupus-like disease and have a lifespan of about five months in males and 14 months in females [87]. When BXSB mice are crossed with other lupus models, such as NZW, MRL, or Fcγ receptor ⅡB-deficient mice, the phenotype induced by the Y chromosome-linked autoimmune accelerator (Yaa) locus is exacerbated [87,100]. Male mice are more severely affected by Yaa, which correlates with the duplication of *Tlr7* and 16 other genes. Furthermore, the sole overexpression of TLR7 can induce autoantibody production, glomerulonephritis, and splenomegaly [99]. TLR7.1 Tg mice express TLR7 at levels 8 to 16 times higher than those in C57BL/6J mice, and approximately 50% of TLR7.1 mice die by six months of age [99]. There are also reports of human SLE caused by gain-of-function mutations in *TLR7*. Further, introducing the TLR7^Y264H^ variant into mice can recapitulate lupus phenotypes [101]. Inducible TLR7-dependent SLE models can be generated by the topical application of TLR7 agonists, such as imiquimod cream (IMQ) or resiquimod, three times a week [102]. These models show no gender bias, and after eight weeks of treatment, glomerulonephritis and autoantibody production are observed [102].

GF or Antibiotics Studies: The application of antibiotics to (NZW × BXSB) F1 mice and TLR7.1 Tg mice rescues mortality rates and reduces autoimmune signatures such as autoantibody production, cytokine levels, and weight loss [4,5]. The mortality rate in GF C57BL/6 mice receiving topical IMQ is significantly lower than in SPF C57BL/6 mice [5].

Dysbiosis: In (NZW × BXSB) F1 mice, *E. gallinarum* translocates from the intestine to the liver and induces autoimmune disease [4]. The translocation of *E. gallinarum* to the liver possibly induces the expression of endogenous retrovirus glycoprotein 70 (ERV gp70) and β_2_-glycoprotein I (β_2_GPI). The immune response to these proteins can drive lupus nephritis in lupus-prone mice via TLR7 and autoimmune thrombi, respectively [4,103,104]. Moreover, *E. gallinarum* RNA can induce type I IFN and other pro-inflammatory cytokines in hepatocytes and dendritic cells [4]. *E. gallinarum* also impacts the expansion of Th17 and T_FH_ cells, which are crucial for systemic antibody production. AHR signaling is involved with the Th17- and autoantibody-inducing effects of *E. gallinarum* [4]. Both TLR7.1 Tg and IMQ-treated C57Bl/6 mice exhibit increased levels of *L. reuteri*, *Desulfovibrio,* and *Rikenellaceae* in their feces. Only *Lactobacillus* species, particularly *L. reuteri*, are observed to translocate to the liver, spleen, and mesenteric lymph nodes [5].

FMT or Colonization Studies: Administration of *L. reuteri* isolated from TLR7.1 Tg mice treated with IMQ to SPF C57BL/6 mice, as well as *L. reuteri* monocolonization followed by IMQ treatment, exacerbated systemic inflammation through the accumulation of pDCs and upregulated type I IFN expression [5].

### 4.4. Triple Congenic (TC) Mice

Description of the Model: In 1994, three recessive loci strongly associated with SLE phenotypes in NZM2410 mice on the C57Bl/6 background, known as *Sle1–3*, were identified [85]. *Sle1* mediates the loss of tolerance to nuclear antigens; *Sle2* reduces the activation threshold of B cells; and *Sle3* causes the dysregulation of CD4 T cells [105]. B6.*Sle1.Sle2.Sle3* mice (triple congenic, or TC, mice) exhibit complete penetrance of fatal glomerulonephritis, resulting in higher mortality rates than NZM2410 mice [105]. This is thought to be due to the absence of the suppressive effect of *Sle4/Sle1* in TC mice [84]. GF TC mice exhibit a sex bias, with female mice showing greater spleen enlargement, higher levels of C3 and Ig deposits in the kidneys, and higher antinuclear antibody scores than male mice [106].

GF or Antibiotics Studies: The application of antibiotics ameliorates autoimmune features in TC mice, such as reducing lymphoid tissue expansion and delaying the onset of anti-dsDNA IgG production [107]. A comparison of TC mice under SPF and GF conditions revealed that splenomegaly, C3 deposition in the kidney, and antinuclear antibody levels are significantly influenced by microbiota and sex bias. However, C3 deposition in the kidney is not significantly affected by either microbiota or sex [106]. This research indicates that the microbiota contributes to sex-dependent immune responses and plays a role in suppressing disease progression in male mice. However, the specific responsible microbes have not been defined [106].

Dysbiosis: *Prevotellaceae*, *Paraprevotella,* and *Lactobacillus* are enriched in the feces of TC mice with autoimmune symptoms compared to C57Bl/6 mice. In contrast, there is no difference in *Prevotellaceae*, *Paraprevotella*, and *Lactobacillus* abundance between young TC mice and C57Bl/6 mice before disease onset [107].

FMT or Colonization Studies: Fecal transfer from TC mice to C57Bl/6 GF mice induces anti-dsDNA antibodies, increases fecal and serum IgA levels, and activates the immune response in mesenteric lymph nodes compared to control mice [107]. It also causes the upregulation of the lupus susceptibility genes *Interferon Regulatory Factor 7 (Irf7)* and *C-terminal Src Kinase (Csk)* [108]. Fecal transfer from young TC mice does not induce autoimmunity, suggesting that aberrant microbiota can accelerate disease progression but is not a trigger for the disease [107].

As discussed above, mouse models provide valuable insights into understanding the precise mechanisms microbes use to shape host immune processes. Since genetic background significantly impacts the microbiome in mouse models, as demonstrated in Table 2, evaluating the consistency of findings with research conducted on human samples is crucial. In mouse lupus models, mice with more specific genetic modifications, such as TLR7.1 Tg and TC mice, may explain how aberrant genetics influence host–microbe communication. On the other hand, since SLE is a multifactorial disease and environmental factors play an essential role in its development, analyzing both traditional and novel lupus models with more discrete genetic changes remains equally important.

## 5. Modulation of Intestinal Processes by Lupus Pathobionts

### 5.1. Enterococcus gallinarum

*E. gallinarum* is a Gram-positive, anaerobic intestinal commensal occasionally found in humans and animals [109]. It exhibits intrinsic resistance to low-dose vancomycin due to the presence of the *vanC* gene and can act as an opportunistic pathogen in humans [110,111]. Although the colonization and infection rates of *E. gallinarum* are lower than those of representative enterococci, such as *E. faecalis* and *E. faecium*, they have been increasing worldwide [109].

*E. gallinarum* evolves to adapt to intestinal luminal and mucosal niches [8]. In GF C57BL/6 mice monocolonized with *E. gallinarum* isolated from fecal samples of (NZW × BXSB) F1 hybrid mice, *E. gallinarum* adapts to these niches through genetic mutations. Translocation of *E. gallinarum* to mesenteric lymph nodes and the liver has been observed in these mice. Notably, mutations observed in liver-translocated *E. gallinarum* are also enriched in populations within the mucosa, indicating that liver-translocating strains originate from mucosa-adapted *E. gallinarum*. Liver-translocated *E. gallinarum* cannot sustain itself in the liver for an extended period and is continuously replenished from the intestine. *E. gallinarum* enriched in fecal samples primarily resides in the intestinal lumen, particularly in the colonic lumen. These strains can stimulate mucus production, goblet cell hyperplasia, and the recruitment of intraepithelial lymphocytes. In contrast, liver isolates of *E. gallinarum* can impair the intestinal barrier and cause inflammation in the lamina propria. They possess a thicker layer of capsular polysaccharides, which aids in immune evasion and can translocate to the liver, inducing inflammation [4,8].

Mice monocolonized with *E. gallinarum* isolated from the liver of (NZW × BXSB) F1 hybrid mice exhibit exacerbated autoimmune phenotypes upon imiquimod application compared to mice colonized with fecal isolates. Similar to GF C57BL/6 mice monocolonized with *E. gallinarum*, GF C57BL/6 mice colonized with *E. gallinarum,* together with a nine-species mock microbial community, also show bacterial translocation to the liver. However, this translocation is absent in gavaged SPF C57BL/6 mice. Furthermore, GF C57BL/6 mice monocolonized with *L. reuteri* exhibit bacterial translocation, whereas monocolonization with *Bacteroides fragilis* does not, despite their genetic divergence between small intestinal mucosal and fecal isolates [8]. These findings indicate that the bacterium itself, host genetic background, and other commensals influence bacterial translocation to distal organs. *E. gallinarum* in genetically predisposed hosts disrupts intestinal homeostasis, translocates to distal organs, and initiates inflammation.

### 5.2. Ruminococcus gnavus

*R. gnavus* is an anaerobic Gram-positive bacterium with a high prevalence of approximately 65–90% in healthy adults but at low abundance, typically 0.1–0.3% [112]. While *R. gnavus* has beneficial roles under steady states, such as strengthening the mucosal barrier and metabolizing nutrients [112], recent studies have highlighted an increase in *R. gnavus* in various diseases, such as inflammatory bowel diseases, irritable bowel syndrome, and type 2 diabetes [112].

A complex glucorhamnan polysaccharide synthesized and secreted by *R. gnavus* induces TNF-α secretion by innate immune cells through TLR4 in vitro [113]. The genes responsible for glucorhamnan biosynthesis are found in every sequenced strain of *R. gnavus* [114]. Subsequently, it was revealed that large capsular polysaccharide-positive *R. gnavus*, such as ATCC29149, does not induce an immune response. In contrast, capsule-negative *R. gnavus* induces proinflammatory responses in vitro, and the gavage of capsule-negative *R. gnavus* into C57BL/6N mice causes inflammation in lamina propria [114]. Additionally, the host’s state, such as the potential for lysozyme production, can influence whether *R. gnavus* is pathogenic or tolerogenic [115].

In SLE patients, *R. gnavus* is five-fold more abundant, and in active patients, it is eight-fold more abundant than in healthy controls. Patients with a history of renal involvement show even more significant expansion of *R. gnavus* compared to those without it [6]. A study using human samples demonstrated that SLE patients have IgG antibodies that recognize cell wall lipoglycans from CC55_01C, a capsule-positive strain [114], whereas healthy controls did not [6]. The fecal abundance of *R. gnavus* correlates with serum IgG anti-CC55_01C antibody levels, a relationship not observed in control subjects. Additionally, normalized serum IgA anti-CC55_01C antibody levels are significantly higher in SLE patients. Furthermore, lupus-associated autoimmune IgG antibodies targeting native DNA were shown to cross-react with epitopes on the CC55_01C strain [6]. Using mouse models, the authors further investigated the association between *R. gnavus* strains and impaired intestinal barriers [80]. They examined litters of GF C57BL/6 mice monocolonized with three strains of *R. gnavus*: one lipoglycan-negative strain (ATCC29149) and two lipoglycan-positive strains (CC55_01C and an SLE patient-derived strain). The study revealed that lipoglycan-positive *R. gnavus* strains induce intestinal barrier dysfunction, especially in female mice, at least partially through zonulin upregulation. The translocation of *R. gnavus* DNA into the mesenteric lymph nodes was also observed. Consistent with human studies, colonization with the SLE-derived *R. gnavus* strain leads to elevated serum levels of IgG anti-native DNA autoantibodies [80]. Many *R. gnavus* strains express B-cell superantigens, which may further explain why *R. gnavus* plays a critical role in the pathogenesis of SLE [116,117].

Some studies show that *R. gnavus* ameliorates mouse disease phenotypes by modulating the production of short-chain fatty acids (SCFAs). Supplementation with 2-fucosylated oligosaccharides ameliorates the disease phenotype of *Il10* knockout mice through the expansion of *R. gnavus* ATCC29149, accompanied by an enhanced concentration of propionate [118]. Oral administration of *R. gnavus* ATCC29149 also ameliorates a mouse atopic dermatitis model by expanding Tregs in mesenteric lymph nodes and skin-draining lymph nodes, along with increased butyrate levels. The functions of *R. gnavus* differ depending on the strains, whether they are capsule-positive or capsule-negative, interaction with other microbes, and the host’s condition.

### 5.3. Bacteroides thetaiotaomicron

*B. theta* is a Gram-negative, anaerobic bacterium and a prominent component of the intestinal microbiome. *Bacteroides* species, including *B. theta*, are generally symbiotic commensals in the intestine [119]. The mucus layer is the first physical barrier against microorganism invasion in the large intestine [58,120]. *B. theta* contributes to intestinal homeostasis by strengthening mucosal barrier formation and regulating the host inflammatory responses [121,122,123,124]. *B. theta* can degrade and utilize various glycans, releasing SCFAs, which contribute to the host’s nutritional state [119,124,125]. It predominantly utilizes dietary glycans, including fibers, but in the shortage of dietary glycans, it shifts to utilizing host glycans [119,126]. Consistent with this observation, a Western diet makes *B. theta* more dependent on utilizing host glycans [127].

Though *B. theta* is known as a symbiont under steady-state conditions, it fails to elicit the anti-inflammatory potential of colonic DCs in patients with Crohn’s disease and ulcerative colitis [128]. Furthermore, fewer circulating DCs produce IL-10 in these patients than in healthy controls [128]. In HLA-B27 transgenic rats, *B. theta* monocolonization leads to chronic colitis, which induces the bacterium to stimulate adaptive immunity [129]. Additionally, during infection with the enteric pathogen enterohemorrhagic *Escherichia coli* (EHEC), *B. theta* enhances EHEC virulence gene expression [130]. These findings suggest that, although *B. theta* generally functions as a symbiont, host pathological states, host genetic background, and interactions with other microbes may contribute to its pathogenic potential.

Another important fact about *B. theta* is that this microbe has a human Ro60 ortholog. Anti-Ro60 antibodies can be positive in several autoimmune diseases, including Sjögren’s syndrome, systemic sclerosis, myositis, and lupus [42]. Although the pathogenic roles of anti-Ro60 antibodies are not fully understood, they are important in SLE, particularly in the pathogenesis of congenital heart block and epitope spreading [131]. No significant difference in *B. theta* abundance is observed in SLE patients compared to healthy controls [7]. However, as mentioned in the “Intestinal dysbiosis in Systemic Lupus Erythematosus” section, this microbe is thought to contribute to SLE pathogenesis through the cross-reactivity of T cells between the commensal Ro60 ortholog and the human Ro60 protein. Gnotobiotic C57BL/6 mice colonized with *B. theta* demonstrated T- and B-cell cross-reactivity between the bacterial Ro60 ortholog and human Ro60. Additionally, IMQ treatment induced higher rates of nephritis-like immune complex deposition in these mice compared to IMQ-treated GF mice [7]. Increased recruitment of APCs and pDCs in the intestine may interact with *B. theta* and contribute to the pathogenesis [7]. Preventing the development of Ro60 reactivity in T and B cells could be a therapeutic target for autoimmune diseases.

### 5.4. Lactobacillus reuteri

*Lactobacillus* species are Gram-positive and facultative anaerobic and widely recognized as probiotics, with *L. crispatus*, *L. gasseri*, *L. salivarius*, and *L. reuteri* being major species in humans [132,133,134]. Studies report beneficial results of using *L. reuteri* as a probiotic against various diseases. [132]. Several mechanisms by which *L. reuteri* suppresses immune activation have been identified, including the activation of AHR and the induction of Tregs [134]. Indole derivatives of tryptophan produced by *L. reuteri* promote the differentiation of intraepithelial regulatory CD4^+^CD8αα^+^ T cells from CD4 T cells in the small intestine by downregulating the transcription factor Thpok [135]. *L. reuteri* induces antimicrobial peptide production from mucosal tissue and produces indole-3-aldehyde. This AHR ligand facilitates IL-22 production from ILC3s, preventing *Candida albicans* colonization and protecting against mucosal inflammation [136]. Treatment of mucosal-like DCs with the culture medium supernatant of *L. reuteri* can upregulate CCR7, essential for migration to mesenteric lymph nodes, and induce FOXP3 and IL-10 expression in Tregs [137]. In NZB/W F1 mice and MRL/lpr F1 mice, the inoculation of *L. reuteri* or a mix of five *Lactobacillus* species, including *L. reuteri,* ameliorates the disease progression [97,138].

Contrary to its well-known probiotic role, *L. reuteri* exacerbates autoimmunity by interfering with metabolic pathways in mouse models of experimental autoimmune encephalomyelitis (EAE) and lupus [5,139,140]. In the EAE model, *L. reuteri* enhances autoimmunity through the upregulation of tryptophan metabolism, and dietary tryptophan depletion suppresses disease activity, consistent with findings in the lupus model of TC mice [108,140]. Apart from tryptophan metabolites, *L. reuteri* inhibits the activity of SCFA-producing bacteria, reduces their metabolites, and exacerbates EAE. Dietary fiber supplementation increases SCFA levels and mitigates the pathogenic role of *L. reuteri* [139]. In TLR7-dependent mouse lupus models, resistant starch (RS) increases SCFA production, reduces the abundance of *L. reuteri*, enhances ileal epithelial barrier function, and prevents translocation to distal organs [5].

In TLR7.1 Tg mice, aerobic and anaerobic non-selective cultures demonstrated bacterial translocation into the mesenteric lymph nodes, liver, or spleen in roughly 90% of the mice [5]. All these were Lactobacillus species, with 75% identified as *L. reuteri*. In contrast, intestinal bacterial translocation into the mesenteric lymph nodes or liver was less than half in TC mice [107]. This may be partly because intestinal barrier integrity was impaired in TLR7.1 Tg but not in TC mice, despite intestinal inflammation in TC mice [5,107]. Although lupus genetic background, strain differences in *L. reuteri*, and interactions with other *Lactobacillus* species or microbiota may differentially impact lupus pathogenesis in mouse models, it is noteworthy that *Lactobacillus* species are increased in the feces of SLE patients compared to healthy controls [5].

**Figure 1:**
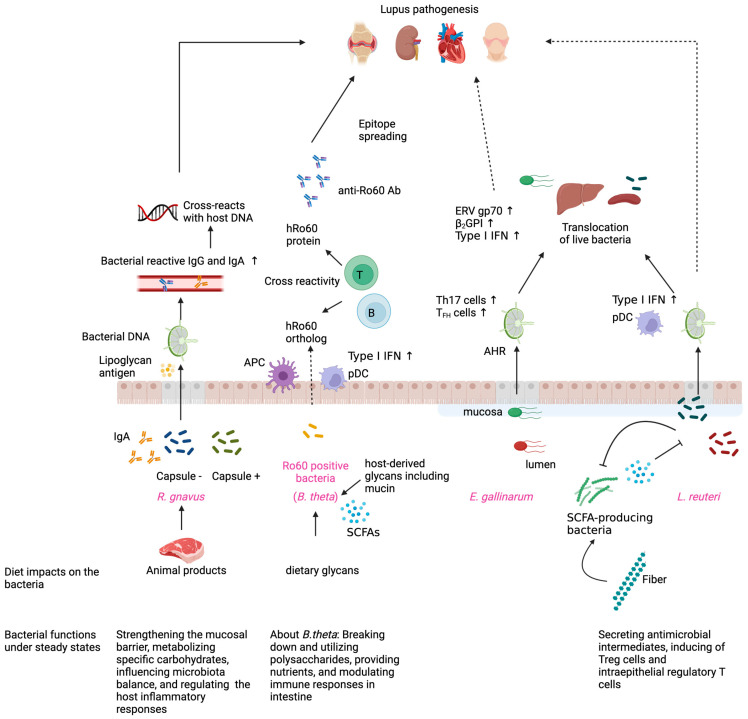
How gut microbes evade and breach the gut barrier and induce host autoimmune responses in systemic lupus erythematosus (SLE).

In summary, the interactions between intestinal bacteria and their hosts are highly dynamic and context-dependent. Host–microbe interactions are shaped by bacterial strains, host genetic backgrounds, environmental factors, and host immune status. While certain bacteria like *E. gallinarum* and *R. gnavus* exhibit pathogenic potential under specific conditions, others such as *B. theta* and *L. reuteri* may shift between beneficial and detrimental roles depending on their environment and host state. They also differ in their preference for colonization niches and their prevalence and occupation rates in steady-state and pathogenic conditions. The host–microbe interactions of well-studied lupus pathobionts are summarized in Figure 1. These findings underscore the importance of maintaining intestinal homeostasis. Furthermore, the differential effects of bacterial strains on autoimmunity and intestinal barrier integrity highlight the therapeutic potential of microbiota-targeted interventions, including dietary modifications and probiotic treatments. Understanding these complex interactions will pave the way for novel strategies to modulate the intestinal microbiome and improve outcomes in autoimmune and inflammatory diseases.

Under steady-state conditions, *Ruminococcus gnavus* has beneficial roles, such as strengthening the mucosal barrier and metabolizing nutrients [112]. However, an increase in *R. gnavus* is observed in various diseases. The expansion of *R. gnavus* is associated with an animal product-rich diet [141]. Large capsular polysaccharide-positive *R. gnavus* strains induce immune responses [114]. Specific strains of *R. gnavus* cause intestinal barrier impairment, and fecal samples from patients with lupus show increased soluble IgA-coated *R. gnavus* [6,80]. *R. gnavus* lipoglycan can leak from the intestinal lumen, and *R. gnavus* DNA has been detected in mesenteric lymph nodes (MLN) in mice with lupus. IgG and IgA anti-*R. gnavus* are increased in lupus patients, and IgG cross-reacts with anti-native DNA. The correlation or inverse correlation of biomarkers with IgG anti-*R. gnavus* suggests that the expansion of *R. gnavus* is associated with lupus nephritis [6]. *Bacteroides thetaiotaomicron* provides nutrients and modulates immune responses under steady-state conditions, and short-chain fatty acids (SCFAs) are produced from dietary and host-derived glycans [124]. *B. theta* (along with other intestinal and skin commensals) has a human Ro60 (hRo60) ortholog. Upon *B. theta* colonization, antigen-presenting cells (APCs) and plasmacytoid dendritic cells (pDCs) increase in the intestine, and T cells and B cells cross-react with the hRo60 ortholog and hRo60 proteins [7]. Anti-Ro60 antibodies are thought to be important for epitope spreading and contribute to SLE pathogenesis. *Lactobacillus reuteri* is commonly considered a probiotic, secreting antimicrobial intermediates and inducing regulatory T cells and intraepithelial regulatory T cells under steady-state conditions [134]. In lupus, *L. reuteri* induces the accumulation of pDCs in MLN, spleen, and Peyer’s patches and upregulates type I IFN expression [5]. In lupus, *Enterococcus gallinarum* induces the production of ERV gp70 protein, β2GPⅠprotein, and type I interferon (IFN) in the liver [4]. In the liver, Anti-ERV gp70 antibodies are thought to contribute to the pathogenesis of lupus nephritis. *E. gallinarum* also expands systemic Th17 and T_FH_ cells, which are crucial for inflammatory responses and antibody production. The aryl hydrocarbon receptor (AHR) pathway is involved in the expansion of Th17 cells. *L. reuteri* and *E. gallinarum* induce gut barrier impairment and translocation to peripheral organs, including the liver and spleen. Remarkably, both pathobionts evolve to adapt to intestinal luminal and mucosal niches, and the *E. gallinarum* that adapted to the mucosal niche translocated to the liver, supporting systemic effects [8]. *L. reuteri* isolated from fecal and liver are genotypically divergent as well. Showing the impact of diet on lupus pathobionts, SCFA production after resistant starch (RS) administration reduces the abundance of *L. reuteri*, enhances ileal epithelial barrier function, and prevents its translocation to distal organs [5]. This figure was created using BioRender (https://www.biorender.com/).

## 6. Current and Future Microbiota-Based Therapies

Although it remains unclear whether the alteration of the microbiota in SLE patients is a cause or consequence of the disease, numerous experiments on lupus-prone mice have demonstrated that interventions targeting the microbiota or its metabolites can improve aberrant immune systems. FMT has progressed to pilot clinical trials. At the same time, interventions such as dietary modification and retinoic acid therapy have shown therapeutic potential in mouse models.

FMT is a therapy in which the recipient receives fecal microbiota precipitate gained from healthy donors’ stool [142]. A pilot clinical trial of FMT has demonstrated its potential as an effective therapy for SLE [143]. The donors comprised seven healthy individuals, including four females and three males, with a mean age of 26.14 ± 2.34 years [143]. Although the number of patients was small, weekly FMT for three consecutive weeks improved SLE Disease Activity Index 2000 (SLEDAI-2K) scores and serum anti-dsDNA antibody levels compared to the baseline. FMT can modulate the intestinal microbiota and influence the host’s immune system. As a result of FMT, an increased production of SCFAs in feces, upregulation of serum S-adenosylmethionine (SAM) levels, genome-wide DNA methylation in peripheral blood, and reduced expression of interferon-related genes in peripheral lymphocytes and NK cells were observed [143,144,145]. Although the total microbiota from healthy donors is currently used in FMT, a combination of specific strains from the microbiota may prove to be a more effective and safer approach. In mouse models, FMT from MRL/lpr mice treated with prednisone to non-treated MRL/lpr mice ameliorate the disease progression without side effects, and the modulation of microbiota caused by prednisone, as well as the decrease in *Ruminococcus* and *Alistipes,* might contribute to the phenomena [146]. Another experiment shows that FMT from (Swiss Webster-Related (SWR) × NZB) F1 (SNF1) mice, a lupus model, given acidic pH water to counterpart mice given neutral pH water, results in disease amelioration [147]. This is because distal ileum samples from SNF1 mice given acidic pH water show decreased levels of Th17/Th9-associated cytokines compared to those from SNF1 mice given neutral pH water [147]. These two studies confirm the effectiveness of FMT in improving the disease condition. These studies propose that FMT can be performed during active disease using stool samples obtained from the same patients when their disease was controlled by therapy.

Regarding diet, the metabolites of fiber and tryptophan play prominent roles in modulating host immune systems. Bacterial metabolites of diet are primarily categorized into SCFAs and tryptophan metabolites [58]. Dietary fibers are a significant source of SCFAs. They are classified into polysaccharides (non-starch polysaccharides [NSPs]), resistant starch (RS), and resistant oligosaccharides (ROs), or soluble and insoluble fibers [148]. Intestinal microbiota ferment soluble fibers to produce SCFAs, such as acetate, propionate, and butyrate [148]. SCFAs serve not only as an energy source but also enhance the functions of intestinal epithelial cells and modulate immune systems in both the intestine and distant organs [58,149,150]. A Western-style diet, characterized by low fiber content, high sugar and fat intake, and high meat protein consumption, contributes to inflammation and chronic disease by reducing SCFAs and upregulating toxic metabolites [148]. Among SCFAs, butyrate is the most extensively studied as an immune system modulator [149]. Butyrate functions via G protein-coupled receptors and inhibition of histone deacetylase (HDAC) activity, regulating the functions of various cell types, including epithelial cells, DCs, B cells, T cells, and innate lymphoid cells [149]. Although SCFAs are thought to suppress aberrant autoimmune diseases, such as multiple sclerosis and type 1 diabetes mellitus, through their immunomodulatory functions, their role in the pathogenesis of lupus remains controversial [150]. Fecal levels of SCFAs are increased in human SLE, potentially due to microbiota alterations, including changes in the F/B ratio [151]. Since SCFAs promote antibody responses and plasma B-cell differentiation [152], they may have multifaceted roles in regulating the pathogenesis of lupus, where autoantibody production is a key feature. Nevertheless, various studies have demonstrated that SCFAs ameliorate lupus pathogenesis [5,153]. In addition to fiber, tryptophan metabolites may interact with the host immune system in SLE. Tryptophan metabolism is complex due to the three main catabolic pathways: the kynurenine pathway, the serotonin pathway, and the indole-3-pyruvate (I3P) pathway. Furthermore, the tryptophan-AHR pathway is regulated by various cell types [154]. In the intestine, indole, produced from tryptophan by microbes under steady-state conditions, enhances barrier function, prevents infections, and supports proper epithelial cell differentiation via AHR [58,154]. In SLE patients, serum tryptophan levels are significantly lower, while kynurenine levels are significantly higher than in healthy controls [155]. Research using fecal samples also reveals altered tryptophan metabolism in SLE [156]. In TC mice, a high-tryptophan diet exacerbates the SLE phenotype, whereas a low-tryptophan diet ameliorates disease progression and improves microbial composition [107]. In (NZW × BXSB) F1 mice, AHR signaling is involved in the expansion of Th17 cells induced by *E. gallinarum*, and an AHR-selective antagonist abrogates the Th17- and autoantibody-expansion [4]. These findings suggest that the altered tryptophan metabolism in SLE contributes to the disease progression.

Retinoic acid modulates the functions of various immune cells and can regulate the immune system through its influence on the microbiome and intestinal integrity [157]. The combination therapy of retinoic acid and prednisolone has shown effectiveness in clinical treatment, although the data are limited [158]. After retinoic acid treatment, MRL/lpr mice show almost normal glomerular histology and reduced proteinuria, although the autoantibody levels are not altered [159]. Retinoic acid restores *Lactobacilli*, depleted in MRL/lpr mice, reversing various lupus-associated metabolic pathways [96]. Furthermore, retinoic acid can induce mucosal-like DCs. In turn, this can induce Tregs, and this phenomenon is enhanced by *L. reuteri* in vitro [137]. Retinoic acid also shows beneficial results in NZB/W F1 mice, reducing glomerular nephritis and serum anti-DNA antibody levels [160].

## 7. Conclusions

SLE is a complex autoimmune disease influenced by multiple factors, including the interactions of immune cells, dietary habits, ethnic backgrounds, and the microbiota. Evidence from human studies highlights intestinal barrier impairments and dysbiosis in SLE patients and provides an association between intestinal health and disease pathogenesis. Several bacterial strains have been implicated in SLE, with growing reports demonstrating their contributions to disease development through mechanisms such as antigen cross-reactivity and microbial translocation. While mouse models offer valuable insights, they also reveal differences in disease manifestations influenced by specific genetic backgrounds. For instance, certain mouse strains exhibit significant intestinal barrier disruption and/or immune activation caused by specific bacterial species, whereas others take advantage of the same species. This underscores the multifaceted feature of SLE, shaped by both host factors and microbial composition. These findings emphasize the importance of understanding how host–microbe interactions contribute to SLE. Hence, further research on the factors that can reverse dysbiosis is essential for developing personalized treatments. In addition to the therapies targeting microbial composition, the supplementation of metabolites from beneficial bacteria, which are decreased in SLE, can represent a more accessible and sustainable therapy. A comprehensive approach combining human studies and mouse models will be crucial to unraveling the intricate mechanisms linking the intestinal microbiota to SLE pathogenesis.

## Figures and Tables

**Table 1:** Altered microbial abundance in SLE patients relative to healthy controls.

Bacterial Taxa	Sample Source	Abundance	References
*Actinomyces massiliensis*	Fecal	Enriched	[70]
Genus *Alistipes*	Fecal	Enriched	[69]
*Alistipes onderdonkii*	Fecal	Enriched	[69]
Genus *Anaerococcus*	Fecal	Enriched	[76]
*Atopobium rimae*	Fecal	Enriched	[70]
Genus *Azospirillum*	Fecal	Decreased	[77]
Genus *Bacteroides*	Fecal	Decreased	[72,76]
*Bacteroides fragilis*	Fecal	Enriched	[70]
*Bacteroides uniformis*	Fecal	Decreased	[6]
Unclassified genus, family *Bifidobacteriaceae*	Fecal	Enriched	[72]
Genus *Bifidobacterium*	Fecal	Decreased	[72]
*Bifidobacterium adolescentis*	Fecal	Decreased	[72]
*Bifidobacterium longum*	Fecal	Decreased	[72]
Genus *Blautia*	Fecal	Enriched	[65]
*Clostridium leptum*	Fecal	Enriched	[70]
*Clostridium species ATCC BAA-422*	Fecal	Enriched	[70]
Genus *Dialister*	Fecal	Decreased	[67,77]
Genus *Eggerthella*	Fecal	Enriched	[67]
Genus *Enterobacter*	Fecal	Decreased	[76]
*Enterococcus gallinarum*	Fecal	Enriched	[4]
Unclassified species, genus *Escherichia*	Fecal	Enriched	[70]
Genus *Eubacterium*	Fecal	Enriched	[67]
Genus *Faecalibacterium*	Fecal	Decreased	[68,72,76]
*Faecalibacterium prausnitzii*	Fecal	Decreased	[68]
Genus *Finegoldia*	Fecal	Enriched	[76]
Genus *Flavonifractor*	Fecal	Enriched	[67]
Genus *Flintibacter*	Fecal	Enriched	[69]
Genus *Fusobacterium*	Fecal	Enriched	[68]
Genus *Gardnerella*	Fecal	Enriched	[76]
Genus *Incertae sedis*	Fecal	Enriched	[67]
Genus *Klebsiella*	Fecal	Enriched	[67]
Genus *Lactobacillus*	Fecal	Enriched	[5,68,72,76]
*Lactobacillus mucosae*	Fecal	Enriched	[68]
*Lactobacillus salivarius*	Fecal	Enriched	[70]
Unclassified genus, family *Lachnospiraceae*	Fecal	Decreased	[72,76]
Genus *Megamonas*	Fecal	Decreased	[72,76]
Genus *Megasphaera*	Fecal	Enriched	[68]
Genus *Odoribacter*	Fecal	Decreased	[65]
Genus *Parabacteroides*	Fecal	Enriched	[69]
Genus *Peptoniphilus*	Fecal	Enriched	[76]
Genus *Phascolarctobacterium*	Fecal	Decreased	[72,76]
Genus *Prevotella*	Fecal	Enriched	[67,72]
Phylum *Proteobacteria*	Fecal	Enriched	[65]
Genus Pseudobutyrivibrio	Fecal	Decreased	[67]
Genus *Rhodococcus*	Fecal	Enriched	[67]
Unclassified genus, family *Rikenellaceae*	Fecal	Decreased	[65]
Unclassified genus, family *Ruminococcaceae*	Fecal	Decreased	[72,76]
*Ruminococcus gnavus*	Fecal	Enriched	[6,69]
*Ruminococcus torques*	Fecal	Enriched	[70]
*Shuttleworthia satelles*	Fecal	Enriched	[70]
Genus *Sneathia*	Fecal	Enriched	[72]
Genus *Streptococcus*	Fecal	Enriched	[68,77]
*Streptococcus anginosus*	Fecal	Enriched	[60,68]
*Streptococcus intermedius*	Fecal	Enriched	[60]
Family *Tannerellaceae*	Fecal	Enriched	[69]
Phylum *Tenericutes*	Fecal	Decreased	[68,69]
Genus *Veillonella*	Fecal	Enriched	[6,68]
*Veillonella dispar*	Fecal	Enriched	[68]
Phylum *Verrucomicrobia*	Fecal	Enriched	[77]
Genus *Actinomyces*	Oral	Decreased	[72]
Unclassified genus, family *Gemellaceae*	Oral	Decreased	[72]
Genus *Granulicatella*	Oral	Decreased	[72]
Genus *Fusobacterium*	Oral	Enriched	[72]
Family *Lactobacillaceae*	Oral	Enriched	[78]
Genus *Lautropia*	Oral	Enriched	[72]
Family *Moraxellaceae*	Oral	Enriched	[78]
Genus *Rothia*	Oral	Decreased	[72]
Genus *Selenomonas*	Oral	Enriched	[72]
Genus *Streptococcus*	Oral	Decreased	[72]
*Streptococcus anginosus*	Oral	Decreased	[72]
Family *Veillonellaceae*	Oral	Enriched	[78]
Genus *Vellionella*	Oral	Enriched	[72]
*Staphylococcus aureus*	Skin	Enriched	[79]
Genus *Anaerococcus*	Vaginal	Enriched	[76]
Genus *Bacteroides*	Vaginal	Enriched	[76]
Genus *Dialister*	Vaginal	Enriched	[76]
Genus *Enterobacter*	Vaginal	Enriched	[76]
Genus *Escherichia-Shigella*	Vaginal	Enriched	[76]
Genus *Faecalibacterium*	Vaginal	Enriched	[76]
Unidentified genus, family *Lachnospiraceae*	Vaginal	Enriched	[76]
Genus *Lactobacillus*	Vaginal	Decreased	[76]
Genus *Peptoniphilus*	Vaginal	Enriched	[76]
Genus *Phascolarctobacterium*	Vaginal	Enriched	[76]
Genus *Porphyromonas*	Vaginal	Enriched	[76]
Genus *Streptococcus*	Vaginal	Enriched	[76]
Genus *Veillonella*	Vaginal	Enriched	[76]

**Table 2:** Altered microbial abundance in fecal samples of lupus-prone mice relative to their control counterparts.

Lupus Model	Control Mice	Age	Gender	Bacterial Taxa	Abundance	References
NZW/B F1 (post)	NZW/B F1 (pre)		Female	Genus *AF12* (family *Rikenellaceae*)	Enriched	[65]
MRL/lpr	MRL/Mp	5 wks	Female	Genus *Alistipes*	Enriched	[96]
TLR7.1 Tg; IMQ	C57BL/6	12 wks; 16 wks	Sex-matched	Genus *Anaerostipes*	Decreased	[5]
MRL/lpr	MRL/Mp	17 wks	Female	*Anaerotruncus* sp. *G3 2021*	Decreased	[70]
				*Bacteroides dorei*	Decreased	[70]
				*Bacteroides vulgatus*	Decreased	[70]
				*Bacteroides xylanisolvens*	Decreased	[70]
NZW/B F1 (post)	NZW/B F1 (pre)		Female	Genus *Bilophila* (family *Desulfovibrionaceae*)	Enriched	[65]
MRL/lpr	MRL/Mp	17 wks	Female	Unclassified species, genus *Brachyspira*	Decreased	[70]
TLR7.1 Tg; IMQ	C57BL/6	12 wks; 16 wks	Sex-matched	Family *Clostridaceae*	Decreased	[5]
NZW/B F1 (post)	NZW/B F1 (pre)		Female	Genus *Clostridium*	Enriched	[65]
MRL/lpr	MRL/Mp	17 wks	Female	*Clostridium* sp. *ASF502*	Enriched	[70]
NZW/B F1 (post)	NZW/B F1 (pre)		Female	Genus *Dehalobacterium*	Enriched	[65]
TLR7.1 Tg; IMQ	C57BL/6	12 wks; 16 wks	Sex-matched	Genus *Desulfovibrio*	Enriched	[5]
MRL/lpr	MRL/Mp	17 wks	Female	*Desulfovibrio desulfuricans*	Decreased	[70]
				*Dorea* sp. *5 2*	Enriched	[70]
				Unclassified species, genus *Dorea*	Decreased	[70]
NZW/B F1 (post)	NZW/B F1 (pre)		Female	Genus *Dorea* (family *Lachnospiraceae*)	Enriched	[65]
(NZW × BXSB) F1	C57BL/6	16wks	Male	*Enterococcus gallinarum*	Enriched	[4]
MRL/lpr	MRL/Mp	5 wks	Female	Family *Lachnospiraceae*	Enriched	[96]
MRL/lpr	MRL/Mp	5 wks	Female	Order *Lactobacillales*	Decreased	[97]
MRL/lpr	MRL/Mp	5 wks	Female	Family *Lactobacillaceae*	Decreased	[96]
NZW/B F1 (post)	NZW/B F1 (pre)		Female	Genus *Lactobacillus*	Enriched	[65]
MRL/lpr	MRL/Mp	17 wks	Female	*Lactobacillus johnsonii*	Enriched	[70]
MRL/lpr	MRL/Mp	17 wks	Female	*Lactobacillus murinus*	Decreased	[70]
TC; TLR7.1 Tg; IMQ	C57BL/6	6-12 mo.; 12 wks; 16wks	Female; Sex-matched; Sex-matched	*Lactobacillus reuteri*	Enriched	[5,107]
TC	C57BL/6	6-12 mo.	Female	Genus *Lactobacillus*	Enriched	[107]
MRL/lpr	MRL/Mp	17 wks	Female	*Mucispirillum schaedleri*	Decreased	[70]
				Unclassified species, genus *Odoribacter*	Decreased	[70]
				Unclassified species, genus *Parabacteroides*	Decreased	[70]
TC; TLR7.1 Tg	C57BL/6	6-12 mo.; 12 wks	Female; sex-matched	Genus *Paraprevotella*	Enriched	[5,107]
TC	C57BL/6	6-12 mo.	Female	Family *Prevotellaceae*	Enriched	[107]
TLR7.1 Tg; IMQ	C57BL/6	12 wks; 16 wks	Sex-matched; female	Genus *Prevotella*	Enriched	[5]
TLR7.1 Tg; IMQ	C57BL/6	12 wks; 16 wks	Sex-matched; female	Order *RF39*	Decreased	[5]
NZW/B F1 (post)	NZW/B F1 (pre)		Female	Genus *Oscillospira*	Enriched	[65]
TLR7.1 Tg; IMQ	C57BL/6	12 wks; 16 wks	Sex-matched; female	Family *Rikenellaceae*	Enriched	[5]
MRL/lpr	MRL/Mp	17 wks	Female	Unclassified species, genus *Roseburia*	Enriched	[70]
MRL/lpr	MRL/Mp	5 wks	Female	Family *Ruminococcaceae*	Enriched	[96]
MRL/lpr	MRL/Mp	17 wks	Female	*Ruminococcus torques*	Enriched	[70]
				*Staphylococcus lentus*	Decreased	[70]
TLR7.1 Tg; IMQ	C57BL/6	12 wks; 16 wks	Sex-matched	Genus *Turicibacter*	Decreased	[5]

IMQ: C57BL/6 mice topically treated with imiquimod cream (IMQ) from 8 to 16 weeks of age. NZW/BF1 (pre) refers to 10, 14, and 18 weeks of age, while NZW/BF1 (post) refers to 23, 28, and 33 weeks of age. The disease onset in NZB/W F1 mice is around seven months of age. wks, weeks; mo., months.

## Data Availability

No new data were created or analyzed in this study.
